# Supplementary material for: Patterns of Intron Gain and Loss in Fungi
Source: PLoS Biol. 2004 Nov 30;2(12):e422. doi: 10.1371/journal.pbio.0020422 (PMC532390; doi:10.1371/journal.pbio.0020422)
Supplement: Table S1 — Also available at http://genes.mit.edu/NielsenEtAl/. (4.3 MB ZIP). [file pbio.0020422.st001.zip › NielsenEtAl/html/995.html]

AN3013.1.NCU02233.1.MG00882.1.FG06325.1


```
 CLUSTAL W (1.82) Multiple Sequence Alignments - Introns Inserted


Sequence 1: NCU02233.1	770 aa
Sequence 2: FG06325.1	795 aa
Sequence 3: MG00882.1	776 aa
Sequence 4: AN3013.1	763 aa
Alignment Length: 812 aa
Number Identitical Residues: 338 aa
Alignment Score (without introns) 18916


MG00882.1 	MAPLRLLIKDGGFLDQHGRQVILRGINVAGDAKFPTTPDGRSHIAKDFFDGDNIDFRERP
NCU02233.1	MAGFRLTIENGSFRDVHGRQITLRGINVAGDAKYPNKPEQPSHVGENFFDGDNVKFTGRP
FG06325.1 	MSSFRLLIEDGQFRDGHGRQVVLRGINLAADAKLPSEPDQPSHIPTDFFDGDNVSFHQRP
AN3013.1  	MASLRLRIDGARFKDPQNREITLRGINVAGEAKYPKSPNVPSNVADNFFDADNVSFVGRP
          	*: :** *... * * :.*:: *****:*.:** *. *:  *::  :***.**:.*  **

MG00882.1 	FPQSEAHVHFARLKRFGYNTIRYIFTWEAIEAAGPGIYDEEWIQHTIEILRIAKNYGFYI
NCU02233.1	FPKEEAHLHFSRLKRFGYNTIRYVFTWEAIEAAGPGIYDEEWIQHTIDVLRVAKRYGFYI
FG06325.1 	FPKEDARSHFSRLRRYGFNTIRYIFTWEALEAAGPGKYDEDFIQHTIDILRIAKEYGFYI
AN3013.1  	FSLDEAHTHFSRLWKLGYNTIRYMFTWEAIEHEGPGKYDDEWISFTIEVLRIAKQYQFYV
          	*. .:*: **:** : *:*****:*****:*  *** **:::*..**::**:**.* **:

MG00882.1 	FMDPHQDV0WSRFSGGSGAPMWTLYAAGLNPEAFAATEAAIVHNTYPDPANFPKMIWSTN
NCU02233.1	FMDPHQDV0WSRFSGGSGAPMWTLYAAGLNPQSFAATEAAIVHNVYPEPHNFPKMIWSTN
FG06325.1 	FMDPHQDV~WSRFTGGSGAPLWTLYACGLNPQSFAATEAAIVHNTYPNPDEFPKMIWSTN
AN3013.1  	FMDPHQDV0WSRLSGGSGAPAWTLYAAGLDPRGFKKTQAALVQNTWDDPAKFPKMIWATN
          	******** ***::****** *****.**:*..*  *:**:*:*.: :* :******:**

MG00882.1 	YYRLAAATMFTLFFAGKDFAPKCIIDGVNIQDYLQGHFMKAISHLAMRIHEAGDIENEVV
NCU02233.1	YYRLAAATMFTLFFAGRDFAPKCIIDGVNIQDYLQDHFLRACAHLAQRIHEAGDIENDVV
FG06325.1 	YYRLAAGTIFTLFFAGKDFAPKCIIDGVNIQDYLQDHFMKACGQLAQRIHEAGDLEDVVV
AN3013.1  	YTRLVCQTMFTLFWAGRDFAPKAIIDGMNIQDYLQSHYISACRYFAQKIHDAGDLENDVV
          	* **.. *:****:**:*****.****:*******.*:: *   :* :**:***:*: **

MG00882.1 	FGWETLNEPNKGMIGYEDISVIPKEQALKKGTSPTIWQAMLTGLGRSTEVDTWDMGSLGP
NCU02233.1	FGWESLNEPNKGMIAYEDISVIPKEQNLKKGTCPTIWQTILTGSGRAVEVDTWDMGGMGP
FG06325.1 	IGWESMNEPNKGMTGYKDLTVIPKEHPLKKGTCPTMWQTMLTGMGRACEVDTWEMGGLGP
AN3013.1  	IGWESLNEPQRGLIGYQDISVIPPEQQLQLGTSPTAFQAMLTGSGRACEEPIWGFGGFGP
          	:***::***::*: .*:*::*** *: *: **.** :*::*** **: *   * :*.:**

MG00882.1 	YKVGRTLVDPKGEIAWLPADYDESRYKYTRDPGWKLGECLWAQHGVWDPATDKLLRKDYF
NCU02233.1	YKVGRALIDPSGEQAWLPADYDESRYGYKRDPGWKLGQCIWAQHGVWDPATDSLLKKDYF
FG06325.1 	YKTGSTLVDPHGEIAWLPEDYDDSRYGWKRDPGWKLGQCIWAQHGVWDPETDTLLRKDYF
AN3013.1  	YQSGRELVDPEGETAWLPVTYDDTKYGWTRDPNWKLGECLWAQHGVWDPATDELLQKDYF
          	*: *  *:** ** ****  **:::* :.***.****:*:********* ** **:****

MG00882.1 	QKNPRTGEVIDHHAFTNTYFMDYARVYRDTVRQHHKEATLILQPPVLELPPSIKGTAD-D
NCU02233.1	GKHPATGEHVDYPYFSNRYFMDFFRKYRDTIRSIHPNAIILLQGPTMELPPKIIGTPDGD
FG06325.1 	AKNPHTGKLIDYPQFTNTYFMDFWRKYVKTCRTVHQDCIMLMQFPTLELPPEIKGTED-D
AN3013.1  	AKKPRTGEPLDYDKFTNTYFLEHYRAYTEAIRSVWPGAIMLCQPPVMEIPPDIKGSND-D
          	 *:* **: :*:  *:* **::. * * .: *     . :: * *.:*:**.* *: * *

MG00882.1 	ENNMVYAPHYYDGITLMTKSW~K-WWNVDVLGVLRGRYLSPVFAIRVGETAVRNCFREQL
NCU02233.1	DPLLVYAPHWYDGITLMTKKW~NRVWNVDVIGILRGKYWSPAFGIKIGETAIRNCFKNQH
FG06325.1 	DPRMAFTPHYYDGITLMTKHW~NSTWNVDVVGVLRGKYWHPALAIRIGETAIRNCLRDQL
AN3013.1  	DPNMVHAVHYYDGLTLMSKHW2NRLYNVDVIGVLRGKYLTPAFGVKIGETAIRNCLRDQL
          	:  :..: *:***:***:* * :  :****:*:***:*  *.:.:::****:***:::* 

MG00882.1 	AAIRQEGLDYMGNHPCILTEFGIPYDMDNKAAYKSGNYSSQSAAMDANHFAVEGAPMEGY
NCU02233.1	ATMRQEGLDYIGNHPCVMTEFGIPYDMDDKNAYKTGDYSSQSAAMDANHYGVEGAGLEGY
FG06325.1 	ATLRQEGLDRTGKHPCVLSEFGIPYDMDDKKAYKTGDYSSQSAAMDANYFAVEGSQIEGH
AN3013.1  	KFLRDESFRYMGNHPLIFTETGIPYDMDDKYAYKTGDYSSQISAMDACHYALEGSSSNGF
          	  :*:*.:   *:** :::* *******:* ***:*:**** :**** ::.:**:  :*.

MG00882.1 	TLWVYCAK0---NDHEYGDQWNGEDLSIYSLDDKALPVSALPPAMGEIGQSTASLVKESS
NCU02233.1	TLWLYMTK0---NDHELGDQWNGEDLSIFSVDDKLLPESPVP----KSHSRDGSSSSIAT
FG06325.1 	CLWTYCAR~---NDHVRGDFWNGEDLSILSLDDTPLPDSPLP----EYSQSTLDLARTAT
AN3013.1  	TLWVYTAK0LLQNDHEWGDQWNGEDLSIYSVDDLELPSGKFL------DHGSASRLDHQS
          	 ** * ::   .***  ** ******** *:**  ** . .            .     :

MG00882.1 	NQEGKLEQSGVEDGTPITPTNLRQTLTSPSISAVPSSSAPEIKNTPGYRAAEAFVRPSPV
NCU02233.1	PTGTKDDD--LDDDSSVTPANIKRTLTNPSISSVSTQRQPELTNSPGYRAAEAYVRPAPI
FG06325.1 	ATNGKKD---VADDRNVTPDNLKRTLTNPSISSAPSTKDPQLTNAPGYRAAEAYVRPTPT
AN3013.1  	PSYSSSHS--HNSNQQISPRNLKDAIKTPSISSQVSQLD---EDRLGYRAAEAYLRPSPI
          	    . ..    ..  ::* *:: ::..****:  :       :  *******::**:* 

MG00882.1 	AVAGSISKYGFDLHKCEFHLEIRAAKAVQTSSDGNPTVVFLPEYHFPKDDCVIEVSSGKW
NCU02233.1	ATAGTVKKYGFDLRSCQFHVTIQAPEAAKPDT---PTVVFLPDYHFPKDACQVEVSSGKW
FG06325.1 	AVYGDVVSTGFDLRQCTYVLKVKAPKAAPDDA---PTIVFLPEYHFPKDQCDVAVSSGKW
AN3013.1  	VTNGLVSEYVFDLRNCSFSMSLVGNRAISGQD--TPTEIFLPEFHFPETHTEVNISSGEW
          	.. * : .  ***:.* : : : . .*   .   .** :***::***:    : :***:*

MG00882.1 	EIGSDDSEG~AL-----VQRFRWWHGEGD-QSLKI~KGVVRTHNVREDGTGADGDVGYYQ
NCU02233.1	EIRSDEEET~TP-----LQKLRWWHGEGE-QTLRV~TGVVKQVNG---NSSEGAEVGYYD
FG06325.1 	ELSIDDEEG1SEEDAGYLEQCQQGYGFNFGSCSVI2TYTVSFLAH---GRLATLRLGNFD
AN3013.1  	LIDHVELRD~VG-----VQRLRWWHPEGD-QSIKI~LGVKRKGGA--LAAPSAEDVSYLE
          	 :   : .         ::: :  :  .  .   :   .                :.  :

MG00882.1 	AVQT------------------VVSQCSIM-------
NCU02233.1	QVFN--------------QAKGFLDACVIM-------
FG06325.1 	IMKTTLLNKLRSISMPAIQTMPSHYDCTTMFLPLSAE
AN3013.1  	QCQT--------------------GGCIVM-------
          	   .                      *  *
```
